# Enhanced Yield of Large-Sized Ti_3_C_2_T_x_ MXene Polymers Nanosheets via Cyclic Ultrasonic-Centrifugal Separation

**DOI:** 10.3390/polym15061330

**Published:** 2023-03-07

**Authors:** Kun Hou, Yafeng Yang, Hu Zhou, Xiangmeng Chen, Shengbo Ge

**Affiliations:** 1 Key Laboratory of Theoretical Organic Chemistry and Functional Molecules, Ministry of Education, School of Chemistry and Chemical Engineering, Hunan University of Science and Technology, Xiangtan 411201, China; 2 Henan Province Engineering Research Centre for Biomass Value-Added Products, School of Forestry, Henan Agricultural University, Zhengzhou 450002, China; 3 Jiangsu Co-Innovation Center of Efficient Processing and Utilization of Forest Resources, International Innovation Center for Forest Chemicals and Materials, College of Materials Science and Engineering, Nanjing Forestry University, Nanjing 210037, China

**Keywords:** Ti_3_C_2_T_x_ MXene, yield, polymers nanosheets, cyclic ultrasonic-centrifugal separation

## Abstract

Water pollution has spurred the development of membrane separation technology as a potential means of solving the issue. In contrast to the irregular and asymmetric holes that are easily made during the fabrication of organic polymer membranes, forming regular transport channels is essential. This necessitates the use of large-size, two-dimensional materials that can enhance membrane separation performance. However, some limitations regarding yield are associated with preparing large-sized MXene polymer-based nanosheets, which restrict their large-scale application. Here, we propose a combination of wet etching and cyclic ultrasonic-centrifugal separation to meet the needs of the large-scale production of MXene polymers nanosheets. It was found that the yield of large-sized Ti_3_C_2_T_x_ MXene polymers nanosheets reached 71.37%, which was 2.14 times and 1.77 times higher than that prepared with continuous ultrasonication for 10 min and 60 min, respectively. The size of the Ti_3_C_2_T_x_ MXene polymers nanosheets was maintained at the micron level with the help of the cyclic ultrasonic-centrifugal separation technology. In addition, certain advantages of water purification were evident due to the possibility of attaining the pure water flux of 36.5 kg m^−2^ h^−1^ bar^−1^ for the Ti_3_C_2_T_x_ MXene membrane prepared with cyclic ultrasonic-centrifugal separation. This simple method provided a convenient way for the scale-up production of Ti_3_C_2_T_x_ MXene polymers nanosheets.

## 1. Introduction

Water pollution from sewage and wastewater produced by industries harms the environment and human health [1,2]. Numerous treatment methods have been adopted for treating pollution and purifying water. Flocculation [3], ultrasonic [4], chlorination [5], adsorption [6], ozonation [7,8] and membrane separation technology [9,10] are some of the methods used in sewage treatment plants for the purification of water. Among the many wastewater treatment processes, membrane separation technology has gained unprecedented development and a good reputation due to its easy operation, reasonable energy consumption, extension possibilities, small footprint and reduction in secondary pollution [9,11]. For example, preparing asymmetric polymer membranes with the phase inversion method is one of the mature methods currently used to produce industrial membranes [12]. This method prepares structures that contain finger-like pores using wet-phase inversion [13,14]. This kind of membrane plays a major role in the separation performance of the epidermal layer, which is easy to form irregular transmission channels but difficult to achieve accurate size screening. In addition, the separation membranes prepared with the electrostatic spinning process [15,16] and the sol-gel method [17,18,19] also result in irregular transmission channels. More importantly, membranes prepared with organic materials may deform easily in the actual separation process, changing their pore size and ultimately affecting the stability of permeate quality. Therefore, in the field of membrane separation, a regular and stable transmission channel is often needed to improve the precise selectivity [20,21]. A significant amount of work has focused on designing and forming regular transmission channels.

Membrane design is a direct application field of nanomaterial research. Two-dimensional materials play an important role in wastewater treatment due to their excellent mechanical properties, ultra-thin layered structure and unique chemical properties [22,23]. In this regard, two-dimensional layered films are attractive because of their regular and stable layered transport channels. Layered separation membranes, such as those prepared from graphene-family materials [24], exfoliated hexagonal boron nitride (h-BNs) [25], layered double hydroxides [26,27], transition metal dihalides (TMD) [28] and transition metal carbides, nitrides or carbonitrides (MXenes) [29], have excellent and precise separation selectivity in molecular or ionic separation. The literature indicates that MXene was discovered in 2011 [30], and research in this field is growing significantly for two-dimensional nanomaterials. So far, more than 30 MXenes have been reported [31]. In the MXene family, Ti_3_C_2_T_x_ is the basis of MXene nanomaterials because of the low cost of the precursor Ti_3_AlC_2_ and the easy etching of aluminum [32,33,34]. In the Ti_3_C_2_T_x_, T_x_ stands for the surface termination group (OH, O or F). Each Ti_3_C_2_ monolayer consists of five layers stacked in the order of Ti(1)—C—Ti(2)—C—Ti(1), which can be described as a Ti_6_C octahedron with shared edges formed by three Ti atomic layers splitting with two C atomic layers [35].

At present, there are two main synthesis methods for the preparation of MXenes. The first method involves the selective etching of precursor material (MAX) with an etching agent followed by stripping (top-down process). In contrast, the second is the chemical vapor deposition (CVD) of molecular precursors (bottom-up method). Although the CVD method can produce high-quality two-dimensional MXenes, they have a large transverse size and a few defects [36] that restrict their use for large-scale industrial membrane manufacturing [37]. Therefore, the top-down approach is currently the focus of researchers to synthesize MXene. Compared with the earliest direct use of hazardous hydrofluoric acid etches, the current trend of using hydrochloric acid (HCl) and fluoride salts, including lithium fluoride or ammonium fluoride (NH4F), to generate HF in situ is emerging rapidly and has become a research hotspot [38,39]. This is because these in situ hydrofluoric acid-etched agents are relatively safe and less toxic to the human body and the environment. Further, the etched Ti_3_C_2_T_x_ MXene nanosheets have a higher O/F ratio, which may benefit the MXene polymers nanosheets in terms of better interaction with the water molecules [40]. These findings are evidence of the potential of MXene material wettability regulation and indicate the chemical versatility required to develop membrane technology.

The design of two-dimensional separation membranes with excellent performance not only requires unique surface chemical properties but also the establishment of regular channels on the premise of large-size nanosheets that still urge for better yield [41]. Fortunately, some new strategies have been employed in recent years to increase the yield of Ti_3_C_2_T_x_ MXene few-layer nanosheets. For example, adding an organic intercalation agent (such as DMSO) during stripping improves the stripping effect of the nanosheet [42]. A freeze-and-thaw-assisted approach or hydrothermal intercalation method improves the preparation effect [43,44]. Meanwhile, the size reduction in the parent MAX phase results in a high yield of nanosheets [45]. Although the literature shows these new developments, the actual process is somehow different and does not meet the standard industrial needs. Therefore, a more convenient method is required for scale-up production.

In the current research, a top-down synthesis route was used for the preparation of multilayer Ti_3_C_2_T_x_ MXene. Further, a cyclic ultrasonic-centrifugal separation (CU-CS) method was used to peel off the multilayer MXene solution to prepare micron-scale single-layer MXene polymers nanosheets. The chemical functional groups and elements of Ti_3_C_2_T_x_ MXene were characterized with Fourier transform infrared spectroscopy and X-ray photoelectron spectroscopy. The morphology and size of Ti_3_C_2_T_x_ MXene were characterized with atomic force microscopy (AFM), scanning electron microscopy (SEM) and X-ray diffraction (XRD). The results revealed that the micron-sized MXene polymers nanosheets could be synthesized with the recycling ultrasonic-centrifugal separation method, and the yield could be significantly improved. The current method may be a suitable approach for synthesizing two-dimensional, material-based nanosheets.

## 2. Experiments and Methods

### 2.1. Materials

Sinopharm Chemical Reagent Co. Ltd. Shanghai, China supplied the HCl specified 36.50%. Shanghai Aladdin Biochemical Technology Co. Ltd. Shanghai, China provided lithium fluoride (LiF) with a 99.99% purity (metals basis). Ti_3_AlC_2_ (MAX, purity: ≥80 wt%, particle size: 1–40 μm) was obtained from Nanjing Xianfeng Nano Material Technology Co. Ltd. Nanjing, China. Polyvinylidene fluoride membrane (diameter: 60 mm, aperture: 0.22 μm) was purchased at the Haining City Yanguan Town Xin Ya filter material business department, Haining, China. Deionized (DI) water, specified as >18 MΩ cm, was utilized for all the required experiments.

### 2.2. Preparation of Ti_3_C_2_T_x_ MXene Polymers Nanosheets

**Figure 1:**
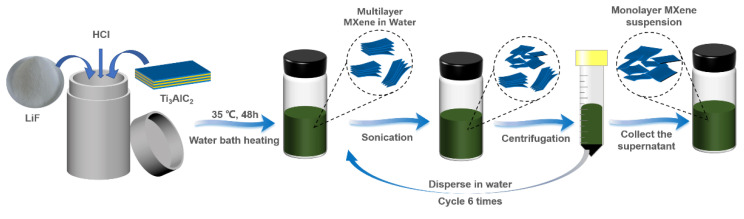
Process Flow Diagram of CU-CS.

For the preparation of the MXene polymers nanosheets, 2.00 g of LiF and 9 M HCl solution (40 mL) were efficiently mixed for 20 min. Afterwards, the Ti_3_AlC_2_ (1.13 g) was slowly added and mixed for 48 h in a 35 °C temperature-maintained water bath. After completing the etching reaction, the obtained suspension underwent centrifugation to separate the sediment. The washing was performed with DI water until it attained a pH of 6. After dispersing the precipitate in water and ultrasonic treatment in an ice bath for 10 min, centrifugation was carried out for 10 min at 3500 rpm to collect the supernatant. The sediment was redispersed in the water, and the ice bath ultrasound was continued for 10 min; then, centrifugation was performed, and the supernatant was collected for a total of 6 cycles (CU-CS method, Figure 1). At the same time, we prepared and collected the samples of continuous ultrasound for 10 min and 60 min.

### 2.3. Characterization

For the structural verification, the functional groups of Ti_3_C_2_T_x_ MXene were characterized by Fourier transform infrared spectroscopy (FTIR, Nicolet5700, Thermo Fisher, MA, USA) in the range of 400–4000 cm^−1^. AFM (atomic force microscopy, VEECO-Multimode, NJ, USA) was used to measure the thickness as well as the size of the single-layer Ti_3_C_2_T_x_ MXene polymers nanosheets in tap mode. XRD (X-ray diffraction, Bruker D8-Advance, Saarbrucken, Germany) was used to estimate the crystallinity of the Ti_3_C_2_T_x_ MXene powder. Field emission scanning electron microscopy (FE-SEM, ZEISS sigma 300, Oberkochen, Germany) was used to characterize the morphology of MAX and Ti_3_C_2_T_x_ MXene. The elemental analysis of Ti_3_C_2_T_x_ MXene was carried out with X-ray photoelectron spectroscopy (XPS, Thermo Scientific K-Alpha, MA, USA). Al Kα ray (1486.6 eV) was the excitation source beam spot: 400 μm; the vacuum degree of the analysis chamber was better than 5.0E-7mBar; the working voltage was 12 kV; and the filament current was 6 mA.

### 2.4. Yield Calculation

In this experiment, the amount of Ti_3_C_2_T_x_ MXene suspension was obtained using the weighing method, and the synthetic yield was further calculated. For this purpose, a dry beaker was taken, and its mass was noted as *M*1 (mg). Then, 10 mL of suspension was placed and dried for 24 h at 100 °C. The water was removed, and after cooling the beaker, it was weighed. Afterwards, the mass was recorded as *M*2 (mg), while the yield was calculated by using Equation (1),

(1) $$Yield=\frac{\left(M2-M1\right)\times V}{10\times M3}\times 100\%$$

where *V* (mL) is the volume of suspension obtained after ultrasonic centrifugal separation, and *M*3 (mg) is the mass of the MAX parent.

### 2.5. Membrane Water Flux Test

In this experiment, the pure water flux (PWF) of the Ti_3_C_2_T_x_ MXene membrane was performed. Initially, nanosheets with different qualities, which were prepared with the cyclic ultrasonic-centrifugal separation method, were placed in 50 mL ultra-pure water and mixed using a stirrer for better uniformity. Then, the membrane was prepared with a vacuum-assisted extraction and filtration method. The supporting membrane was PVDF (effective diameter was 47 mm). The suspension was placed in the solvent filter, and after standing for ten minutes, a transmembrane pressure of 0.2 bar was applied. After the water was completely lost, the membrane was removed and placed for 24 h in a 60 °C-maintained drying oven.

Before the PWF test, the prepared membrane was moistened for 5 min and then assembled in a solvent filter with a certain amount of water and a 0.9 bar transmembrane pressure. The filtrate samples were collected after specifically selected intervals, and the PWF for the prepared membrane was calculated according to Equation (2),

(2) $$F=\frac{M4}{\Delta P\times A\times \Delta t}$$

where *F* (kg m^−2^ h^−1^ bar^−1^), *M*4 (kg), Δ*P* (bar), *A* (m^2^) and Δ*t* (h) represent the PWF, the filtrate mass, the transmembrane pressure, the effective area of the separating membrane and the membrane separation time, respectively.

## 3. Results and Discussion

### 3.1. Composition and Structure Characterization

**Figure 2:**
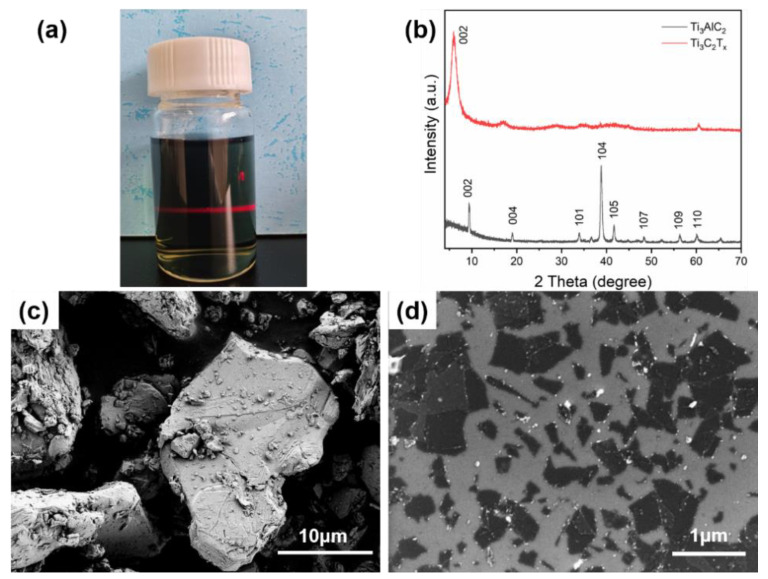
(**a**) The Tyndall effect for the monolayer Ti_3_C_2_T_x_ colloidal solution; (**b**) The XRD patterns for Ti_3_AlC_2_ and Ti_3_C_2_T_x_ and the SEM images of (**c**) Ti_3_AlC_2_ and (**d**) Ti_3_C_2_T_x_.

The relatively mild etching conditions of HCl and LiF were used to etch the aluminum atomic layer in the precursor Ti_3_AlC_2_ MAX phase because very stable M–a metallic bonds connect the MAX phase, and it is very difficult to peel it off with only mechanical shearing. After etching, a single layer of the Ti_3_C_2_T_x_ MXene polymers nanosheets can be synthesized with an easy ultrasonic exfoliation method. As shown in Figure 2a, the obvious Tyndall effect can be observed from the figure [46], so it can be determined that it is a colloidal solution. Therefore, the Ti_3_C_2_T_x_ MXene polymers nanosheets can be preliminarily determined to be successfully prepared. To further investigate whether the single-layer Ti_3_C_2_T_x_ MXene polymers nanosheets were successfully prepared, the parent MAX and the synthesized MXene polymers nanosheets were characterized with XRD. The diffraction peaks at the plane 002 and plane 104 can be observed in the original MAX phase. The diffraction peaks at the plane 104 represent the element aluminum (Figure 2b). After etching, the diffraction peak of Ti_3_AlC_2_ MAX at the 104 plane at 39° does not exist in the spectrum of Ti_3_C_2_T_x_ MXene, indicating that the Al atomic layer was removed by the wet etching. More importantly, the diffraction peak in the 002 plane shifted from 9.7° to a lower angle (5.9°) because of the layered structure of the exfoliated nanosheets stacked on each other [29].

**Figure 3:**
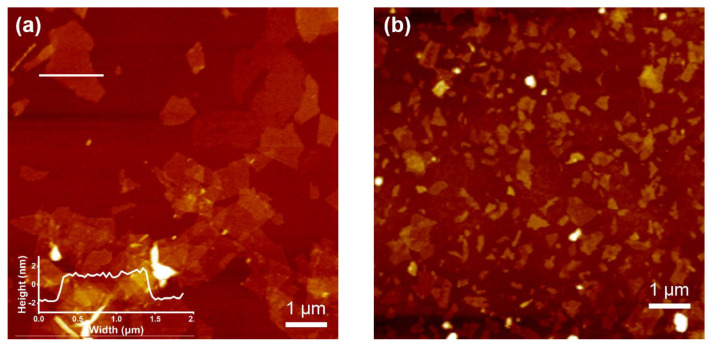
The AFM images of Ti_3_C_2_T_x_ MXene: (**a**) Ultrasound 10 min and (**b**) ultrasound 60 min.

The SEM images revealed that the MXene was peeled off into sheets after etching (Figure 2c,d). After the aluminum atoms in the bulk parent Ti_3_AlC_2_ MAX were etched and ultrasonically exfoliated, Ti_3_C_2_T_x_ MXene showed a single-layer lamellar structure. In this case, the individual lamellae were separated from each other and uniformly dispersed on the silicon wafer, and the nanosheets possessed micro sizes. The synthesized MXene polymers nanosheets possessed a thickness of 1 to 2 nm, indicating that the nanosheets were a monolayer. This is also obvious from the atomic force microscopy (AFM) (Figure 3).

**Figure 4:**
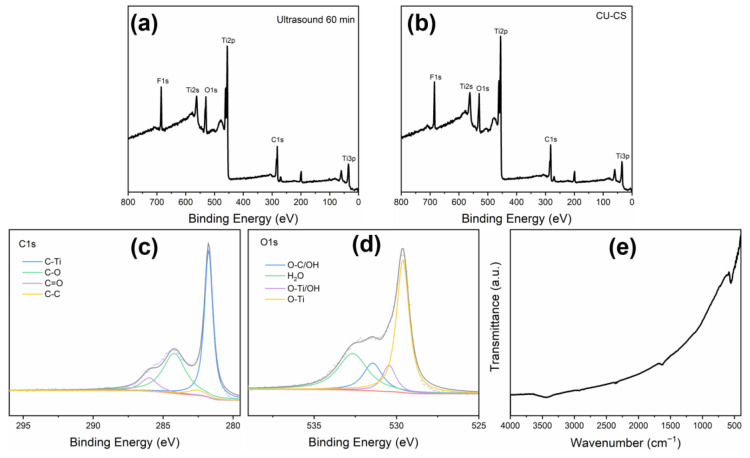
(**a**) The XPS results of the Ti_3_C_2_T_x_ MXene nanosheets obtained with ultrasound for 60 min; (**b**–**d**) The XPS results of the Ti_3_C_2_T_x_ MXene nanosheets obtained with CU-CS; (**e**) The FTIR spectra for Ti_3_C_2_T_x_ MXene.

The XPS helped determine the chemical composition of the Ti_3_C_2_T_x_ MXene nanosheets prepared with different methods. Ti_3_C_2_T_x_ MXene’s XPS measurement spectrum and the corresponding high-resolution spectrum are presented in Figure 4a–d. It was found that the Ti_3_C_2_T_x_ MXene nanosheets prepared with different methods mainly contained C (C1s), F (F1s), Ti (Ti2p) and O (O1s) elements, which also indicates that Ti_3_C_2_T_x_ MXene was successfully etched from Ti_3_AlC_2_ powder. The nanosheets prepared with both methods exhibited almost similar elemental composition, indicating that the ultrasonic method had no effect on the etching of the original MAX phase and improved the stripping efficiency. It is also worth noting that C-Ti (281.7 eV), C-C (282.6 eV), C-O (284.2 eV) and C=O (286.0 eV), and O-Ti (529.6 eV), O-Ti/OH (530.5 eV), O-C/OH (531.5 eV) and H_2_O (532.7 eV) can be observed in the XPS analysis of C1s and O1s (Figure 4c,d) [47,48]. These results indicate the existence of different oxygen-containing functional (OCF) groups in Ti_3_C_2_T_x_ MXene. The FTIR results shown in Figure 4e indicate the functional groups of Ti_3_C_2_T_x_ MXene are presented. It can be observed that the peaks at 556.9 cm^−1^, 1639.8 cm^−1^ and 3444.5 cm^−1^ are the result of the stretching vibration of the Ti-O bond, C-O bond and -OH functional groups, respectively [49]. These results indicate that the prepared Ti_3_C_2_T_x_ MXene nanosheets contain enough oxygen-containing functional groups, which is consistent with the results of the XPS spectra. The Ti_3_C_2_T_x_ MXene nanosheets contain enough OCF groups to be advantageous in membrane separation applications.

### 3.2. Effect of the Ultrasonic Method on the Size and Yield of the Nanosheets

The time of sonication influenced the size of the nanosheets. In the current research work, the effect of continuous ultrasonic time on the size of the nanosheets was determined. As shown in Figure 3, the nanosheets prepared after continuous ultrasound for 60 min possessed a few hundred nanometers. The one prepared using an ultrasonic treatment of 10 min exhibited the micro sizes. This may be because the mechanical stability of single-layer nanosheets is insufficient for long-term ultrasonic treatment. The collision leads to the fracture of the nanosheets that have a high aspect ratio, thus forming smaller nanosheets. This revealed that the size of the prepared MXene polymers nanosheets could be controlled by the ultrasonic time.

**Figure 5:**
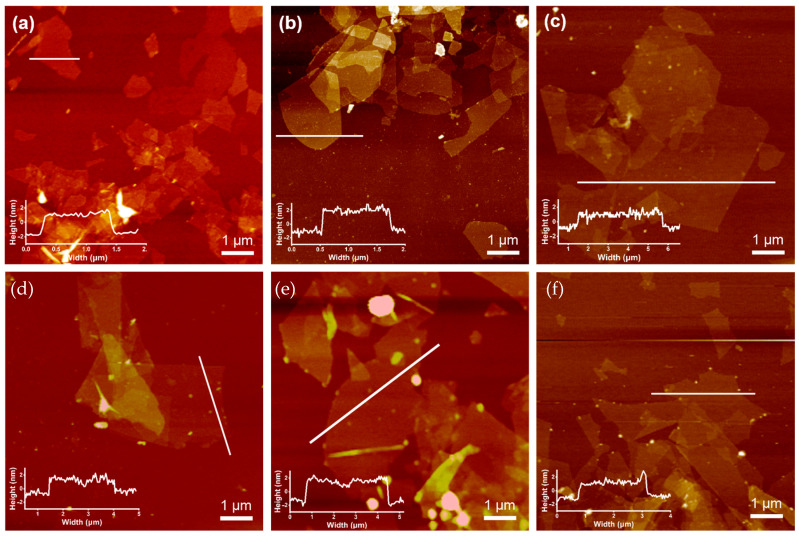
The AFM images of CU-CS Ti_3_C_2_T_x_ MXene: (**a**) 1, (**b**) 2, (**c**) 3, (**d**) 4, (**e**) 5 and (**f**) 6.

We compared the morphologies of the nanosheets prepared with the CU-CS method with different cycles (Figure 5). The AFM images indicated that the micrometer-sized Ti_3_C_2_T_x_ MXene nanosheets were synthesized with six cycles of ultrasonic-centrifugal separation, and all Ti_3_C_2_T_x_ MXene nanosheets possessed a thickness of 1 to 2 nm. It is obvious that a single layer of the Ti_3_C_2_T_x_ MXene nanosheets with a high aspect ratio was prepared each time. The reason behind this could be the long time and high energy required to separate the bulk multilayer Ti_3_C_2_T_x_ MXene during the exfoliation process. After the CU-CS, the exfoliated monolayer Ti_3_C_2_T_x_ MXene nanosheets were separated from the multilayer Ti_3_C_2_T_x_ MXene nanosheets. This prevented the influence of long-term ultrasound on the size of the nanosheets.

**Figure 6:**
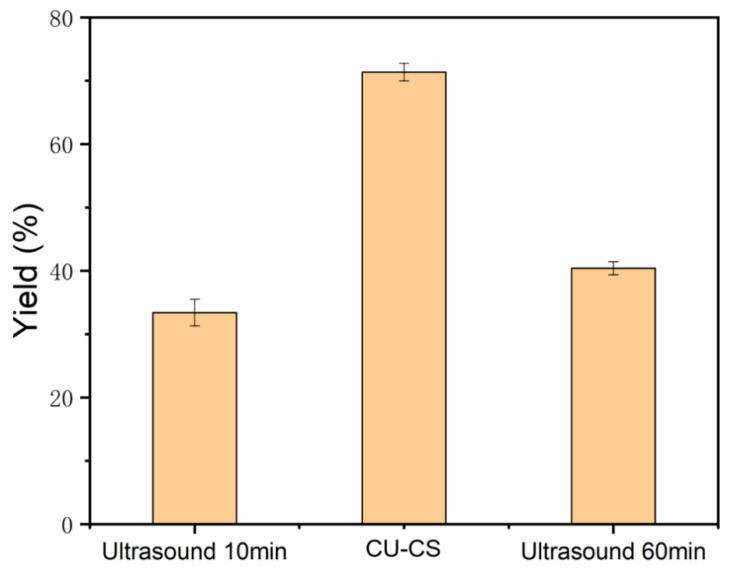
Effect of the ultrasonic method on the yield of monolayer Ti_3_C_2_T_x_ MXene.

In addition, we analyzed the yield of Ti_3_C_2_T_x_ MXene nanosheets synthesized with different ultrasonic methods. The ultrasonic treatment for 10 min resulted in a yield of 33.40%. In comparison, continuous ultrasonic treatment for 60 min yielded 40.39% (Figure 6). Whereas the CU-CS method helped attain a yield of 71.37% for Ti_3_C_2_T_x_ MXene nanosheets, which was 2.14 times and 1.77 times higher than that prepared with continuous ultrasonication for a time of 10 min and 60 min, respectively. The high yield of the nanosheets prepared with the CU-CS method is the participation of the remaining precipitates that are continuously stripped to maximize the yield. In addition, under the same ultrasound time, the yields of cyclic and continuous ultrasound are not consistent, which may be because part of the energy of continuous ultrasound is consumed in the fragmentation of the nanosheets; thus, the multilayer MXene cannot be fully exfoliated. All of these results indicate that the CU-CS method can enhance the yield of large-sized MXene polymers nanosheets. This method could also be beneficial in terms of cost. The CU-CS method only needs to separate the MXene polymers nanosheets exfoliated with ultrasound with centrifugation in time. The operation processes do not require additional equipment in actual production, thus greatly reducing production costs and making commercial production of layered MXene separation membranes possible.

### 3.3. Application of Ti_3_C_2_T_x_ MXene Nanosheets

**Figure 7:**
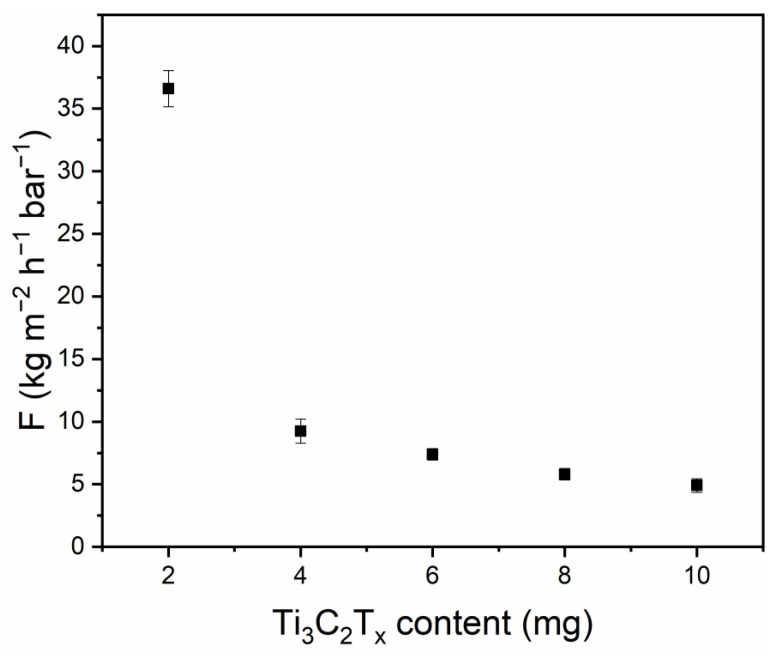
Pure water fluxes of the Ti_3_C_2_T_x_ MXene membranes with different contents.

The Ti_3_C_2_T_x_ MXene nanosheets are widely used in electromagnetic shielding, energy storage, catalysis, and antibacterial and membrane separation fields since the inception of Ti_3_C_2_T_x_ MXene. These nanosheets have attracted much attention for membrane separation applications, especially because they contain reasonable OCF groups that provide hydrophilicity to Ti_3_C_2_T_x_ MXene nanosheets. The PWF of the Ti_3_C_2_T_x_ MXene membrane prepared with vacuum-assisted filtration was also tested (Figure 7). Experiments revealed that, when the content of Ti_3_C_2_T_x_ MXene is 2 mg, the membrane had a PWF of 36.5 kg m^−2^ h^−1^ bar^−1^. With an increase in the amount of Ti_3_C_2_T_x_ MXene, the PWF of the membrane decreased. This is because the massive stacking of Ti_3_C_2_T_x_ MXene increases the water transmission channel, thus enhancing the mass transfer resistance of the membrane. In this case, the PWF of the membrane was found to be 4.9 kg m^−2^ h^−1^ bar^−1^ for 10 mg of Ti_3_C_2_T_x_ MXene. Application-wise, the size of the nanosheet may also influence its performance. One example of these membranes is pressure-driven electric power generation [50] They first prepared two kinds of Ti_3_C_2_T_x_ MXene nanosheets with transverse sizes of 0.7–1.1 μm and 2–3 μm, respectively, and then filtered the different sizes of Ti_3_C_2_T_x_ MXene onto nylon-supported membranes with a simple vacuum-assisted filtration method. It was observed that the membranes with small-size nanosheets responded better to current than those with large-size nanosheets under the same amount of Ti_3_C_2_T_x_ MXene deposition. The reason could be the occurrence of a longer path of ions and water molecules through the large nanosheets. In the field of osmotic evaporation desalination, [51] also prepared Ti_3_C_2_T_x_ MXene membranes using a simple vacuum-assisted filtration method. They used a commercial polyacrylonitrile (PAN) ultrafiltration membrane having a pore size of a few nanometers as a support and then filtered Ti_3_C_2_T_x_ MXene nanosheets with transverse sizes of 0.5 μm and 1–2 μm on the support to form a film. They found that, when the content of Ti_3_C_2_T_x_ MXene was the same, the water flux of the small-size Ti_3_C_2_T_x_ MXene nanofilms (~0.5 μm) was slightly higher than that of the large-size Ti_3_C_2_T_x_ MXene nanofilms (~1–2 μm). After SEM and AFM characterization, the large Ti_3_C_2_T_x_ MXene nanosheets showed a more compact stacking structure. This could also be a reason for the reduction in water flux in the membranes prepared with large-size Ti_3_C_2_T_x_ MXene nanosheets. However, it has to be mentioned that the comparison of these results is in the same Ti_3_C_2_T_x_ MXene content, and the large-size nanosheets are easier to form a compact stack. Under the condition of the same retention rate, the Ti_3_C_2_T_x_ MXene load can be reduced to prepare ultra-thin Ti_3_C_2_T_x_ MXene membranes with faster permeability, and the large-size nanosheets are easier to form regular transmission channels. This also indicates the possibility of precise transport of molecules or ions.

## 4. Conclusions

Combining the top-down chemical etching and the CU-CS methods, an efficient way for synthesizing the large-sized Ti_3_C_2_T_x_ MXene nanosheets was successfully developed. The CU-CS method provides a large space for improving the yield of Ti_3_C_2_T_x_ MXene nanosheets and increases the yield of single-layer Ti_3_C_2_T_x_ MXene nanosheets to a certain extent. CU-CS experiments showed that the yield of Ti_3_C_2_T_x_ MXene nanosheets synthesized with the CU-CS method reached 71.37%, which is 2.14 times and 1.77 times higher than that prepared with ultrasonic treatment for 10 min and 60 min, respectively. More importantly, the size of the single-layer Ti_3_C_2_T_x_ MXene nanosheets synthesized with the CU-CS method was not significantly reduced, and the single-layer Ti_3_C_2_T_x_ MXene nanosheets possessed micron size. In addition, the pure water flux experiments of the membranes showed that the membranes prepared with the CU-CS method had excellent permeability. When the content of Ti_3_C_2_T_x_ MXene was 2 mg, the PWF of the membranes was 36.5 kg m^−2^ h^−1^ bar^−1^. Even when the content of Ti_3_C_2_T_x_ MXene was 10 mg, the PWF of the membrane still maintained a certain amount. Compared with the asymmetric irregular pore size in other polymer separation membranes, the micron-sized Ti_3_C_2_T_x_ MXene polymers nanosheets have great potential in preparing separation membranes with regular transmission channels and provide the premise of yield and quality for the application in the field of membrane separation. Overall, this research provides a new way to develop efficient preparation of single-layer Ti_3_C_2_T_x_ MXene nanosheets, which are expected to be used in large-scale industrial production.

## Data Availability

The data presented in this study are available on request from the corresponding author.
